# Molecular dynamic simulations of *Escherichia coli*l-asparaginase to illuminate its role in deamination of asparagine and glutamine residues

**DOI:** 10.1007/s13205-015-0339-9

**Published:** 2015-12-29

**Authors:** Rajeswara Reddy Erva, Satish Babu Rajulapati, Chandrasai Potla Durthi, Mayuri Bhatia, Madhuri Pola

**Affiliations:** 1 Department of Biotechnology, National Institute of Technology Warangal, Warangal, 506004 Telangana India; 2 School of Information Technology, Jawaharlal Nehru Technological University Hyderabad, Hyderabad, 500085 Telangana India

**Keywords:** l-asparaginase, Molecular docking, Molecular dynamic simulations, Acute lymphocytic leukemia

## Abstract

Acute lymphocytic leukemia (ALL) is an outrageous disease worldwide. l-Asparagine (l-Asn) and l-Glutamine (l-Gln) deamination play a crucial role in ALL treatment. Role of Elspar^®^ (l-asparaginase from *Escherichia coli*) in regulation of l-Asn and l-Gln has been confirmed by the other researchers through experimental studies. Therapeutic research against ALL remained elusive with the lack of information on molecular interactions of Elspar^®^ with amino acid substrates. In the present study, using different docking tools binding cavities, key residues in binding and ligand binding mechanisms were identified. For the apo state enzyme and ligand bound state complexes, MD simulations were performed. Trajectory analysis for 30 ns run confirmed the kinship of l-Asn with l-asparaginase enzyme in the dynamic system with less stability in comparison to l-Gln docked complex. Overall findings strongly supported the bi-functional nature of the enzyme drug. A good number of conformational changes were observed with 1NNS structure due to ligand binding. Results of present study give much more information on structural and functional aspects of *E. coli* l-asparaginase upon the interaction with its ligands which may be useful in designing effective therapeutics for ALL.

**Electronic supplementary material:**

The online version of this article (doi:10.1007/s13205-015-0339-9) contains supplementary material, which is available to authorized users.

## Introduction

Uncontrolled division of cells is defined as cancer. Cancer of white blood cells (WBC), characterized by excessive multiplication of malignant and immature WBC (lymphoblast) in bone marrow is referred to as acute lymphoblastic leukemia (ALL). Methods of treatment of acute leukemia include chemotherapy, radiation therapy, steroids, and intensive combined treatments including stem cell or bone marrow transplants. Chemotherapy is the most preferred way of treatment among the above. Various drugs employed for treatment of ALL include asparaginase, dexamethasone, prednisolone, vincristine, daunorubicin, cyclophosphamide, cytarabine, etoposide, thioguanine, mercaptopurine, hydrocortisone, methotrexate etc.

**Fig. 1:**
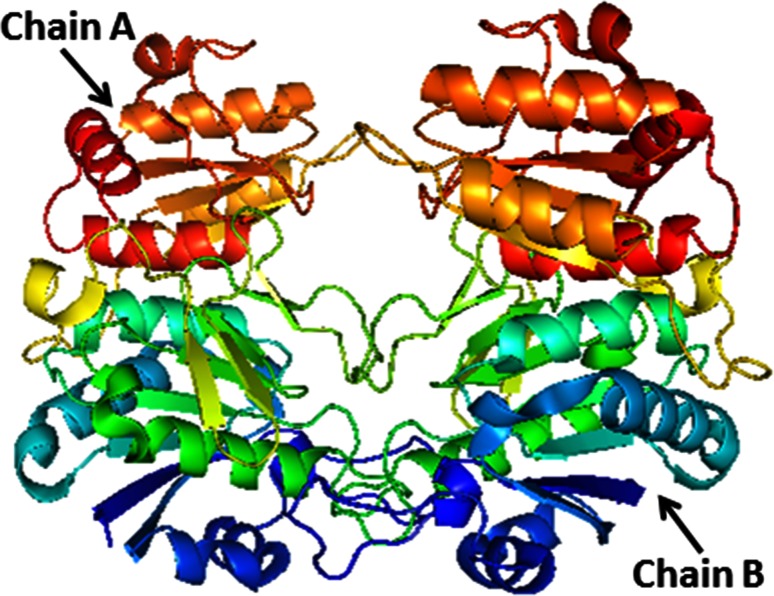
Crystal structure of *E. coli* l-asparaginase (PDB ID: 1NNS)

Recently l-asparaginase has evolved as an important enzyme in growing enzyme industry, owing to its potential use in the treatment of ALL and lymphosarcoma (Story et al. 1993; Verma et al. 2007) and also in food industry to prevent acrylamide formation in fried foods at high temperatures (Pedreschi et al. 2008). l-asparaginase catalyzes the hydrolysis of amide group of side chain in l-Asn to yield l-aspartate and ammonia. By treating patients with l-asparaginase, circulating plasma pools l-Asn levels were effectively depleted in the body (Fig. 1), resulting in the inhibition of protein synthesis followed by inhibition of DNA and RNA synthesis. It causes apoptic cell death of leukemic cells, thereby makes it selective against the leukemic cells without affecting the normal cells (Nandy et al. 1997). As some leukemic cells are unable to synthesize the asparagine synthetase enzyme, they are totally dependent on circulating extracellular l-Asn. Currently l-asparaginase purified from *Escherichia coli* is extensively used in clinical treatment of leukemia which is available in the market with the brand name of Elspar^®^ (PDB ID: 1NNS). The possible side effects reported with Elspar^®^ include severe allergic reactions (rash; hives; itching; difficulty breathing; tightness in the chest; swelling of the mouth, face, lips, or tongue); fever; pain, redness, or swelling at the injection site; symptoms of liver problems (e.g., dark urine, pale stools, nausea, loss of appetite, unusual tiredness, yellowing of the skin, or eyes); symptoms of pancreatitis (e.g., severe stomach or back pain with or without nausea or vomiting); neurological seizures and induction of anti-asparaginase antibodies that inactivate the anti-cancer enzyme (Verma et al. 2007; Heinemann and Howard 1969; Savitri and Azmi 2003). To overcome the toxicity associated with preparations of asparaginase from the current sources, there is a need for identification of a new serologically different enzyme which has the same therapeutic effect. To obtain a better and alternative source of l-asparaginase, there is a huge ongoing interest to screen various organisms from various biodiversities.

Although the l-asparaginase enzyme from guinea pig serum has no l-Glutaminase activity (Tower et al. 1963), bacterial l-asparaginase exhibits its activity with l-Gln as a substrate (Campbell and Mashburn 1969; Campbell et al. 1967; Howard and Carpenter 1972; Roberts et al. 1972; Tosa et al. 1972; Wriston Jr 1971). These two activities have been studied in *E. coli* enzyme preparations (Campbell and Mashburn 1969; Miller and Balis 1969). Upon treating patients of ALL with l-asparaginase, a marked depletion in both extracellular and intracellular glutamine has been observed both in vitro (Bussolati et al. 1995; Uggeri et al. 1995) and in vivo (Ollenschläger et al. 1988; Reinert et al. 2006; Rudman et al. 1971). In many tissues, a severe metabolic stress is caused by Glutamine starvation and is followed by the up-regulation of the expression and/or activity of glutamine synthetase (GS) that obtains glutamine from glutamate and ammonium (Lacoste et al. 1982). Treatment with the anti-tumor enzyme produces a marked increase in GS expression and a stimulation of GS activity. Moreover, in the same cells the inhibition of GS activity abolishes resistance to the cytotoxic effects of asparaginase leading to massive cell death. In those cells that are poorly sensitive to the anti-tumor enzyme, the effects of asparaginase are significantly enhanced by GS inhibition (Tardito et al. 2007). This laid the platform for this current study to understand the molecular information about the enzyme and its interactions with the substrates through docking and testing the stability of the enzyme and docked complexes under physiological conditions by molecular dynamics and simulations methods.

## Materials and methods

### Preparation of ligands and receptor

Ligand molecules l-Asn (C_4_H_8_N_2_O_3_) and l-Gln (C_5_H_10_N_2_O_3_) whose molecular masses are 132.12 and 146.14 g/mol were retrieved from Zinc database with ID numbers 1532556 and 1532526, respectively. Then they were subjected for energy minimization using the MMFF (Merck Molecular Force Field) (Halgren 1996) of VLifeMDS v 4.3 that works based on MM3 force fields until reaching global minima. Crystal structure of l-asparaginase II from *E. coli* was obtained from Protein Data Bank (PDB: 1NNS) (Sanches et al. 2003).

### Molecular docking using Hex 8.0.0, PatchDock and FireDock

Hex is a rigid-body docking tool for use with large molecules such as DNA and proteins. Assuming the ligand is rigid, it computes protein ligand docking using Spherical Polar Fourier (SPF) correlations to accumulate the calculations (Sridhar et al. 2005). Global docking score can be obtained as a function of the six degrees of freedom in rigid-body docking, by scripting expressions for the overlay of pairs of parametric functions (Ritchie 2003; Ritchie and Kemp 2000). The docking score was obtained using the default parameters, and the same was interpreted as interaction energy between the ligand and receptor.

In order to verify the results obtained by Hex, another molecular docking was performed by Patch Dock server by submitting the structures to web server (Schneidman-Duhovny et al. 2005) that works based on shape complementarity principles and again the outcomes were refined with FireDock server (Andrusier et al. 2007; Mashiach et al. 2008) that reshuffles the interface side chains and amends the molecule’s relative orientation. Analysis of ligand binding interactions and docking viability was done based on Fire Dock scores and visualized using Pymol.

### Molecular dynamics and simulations

MD simulations were executed for the apo enzyme, 1NNS—l-Asn (complex 1) and 1NNS—l-Gln (complex 2) docked complexes gained from molecular docking to ratify the stability for anti-cancer enzyme in apo state and bound state with the substrates in dynamic system. Generating both the l-Asn and l-Gln topologies using PRODRG server is the early step in MD simulations (Schüttelkopf and Van Aalten 2004). After defining ligand topologies, MD simulation for apo enzyme and docked complexes was carried using GROMACS 4.6.5 program package under Ubuntu 14.04 operating system. Steepest algorithm using the OPLS force field (Lindahl et al. 2001) was used for energy minimization, dismissing the step when the maximum force is found lesser than 1000 kJ mol^−1^ nm^−1^. To provide an aqueous environment in a system of a cubic box with a size of 1.0 nm and at least 2.0 nm between any two periodic protein images, all the molecules were solvated. With the addition of six Sodium ions, the system was neutralized and periodic boundary conditions were applied in all directions. The cubic interpolation order in Particle Mesh Ewald (PME) simulation method is 4 and the grid spacing for FFT (Fourier spacing) is 0.16. In the neighbor searching method, the short-range neighbor list cutoff of 1 Å is taken commonly for electrostatic interactions and van der Waal interactions. The LINCS (Hess et al. 1997) and SETTLE algorithms (Miyamoto and Kollman 1992) were applied to constrain all bond lengths and geometry of water molecules, respectively, in the system. For 100 ps duration, the two equilibration phases, NVT ensemble with a constant temperature of 300 K, coupling constant of 0.1 ps, and NPT ensemble with constant pressure of 1 bar, coupling constant of 2 ps, were applied for all the molecules. Modified Berendsen thermostat coupling scheme algorithm was employed for both ensembles of equilibration. Once the system equilibration with constant temperature and pressure is done, a 30 ns production MD run was performed to carry out the structural and energy analyses. Run trajectories were obtained and quality assurance of all the molecules was done with GROMACS utilities, namely g_energy, g_rms, g_rmsf and g_gyrate. Hydrogen bonding analysis was done by g_hbond. Using XMGRACE tool, the entire trajectory results were analyzed (Turner 2005).

## Results and discussion

As per the structural description provided by the depositors a total of nine α-helices (54–59; 85–98; 114–124; 147–159; 248–255; 273–284; 308–311; 321–331 and 338–345) and fourteen β-sheets (25–32; 69–78; 104–108; 131–134; 169–172; 175–178; 182–184; 193–195; 202–205; 208–211; 236–240; 260–265; 288–293 and 313–315) are present in it. Based on *interpro* analysis (http://www.ebi.ac.uk/interpro/), N-terminus of 1NNS has two conserved threonine residues with catalytic role.

## Docking with l-asparagine and l-glutamine

Two ligand substrates namely l-Asn and l-Gln were docked into the catalytic site of 1NNS. Dock runs of ligands on the enzyme were performed using HEX 8.0.0 and PatchDock.

**Fig. 2:**
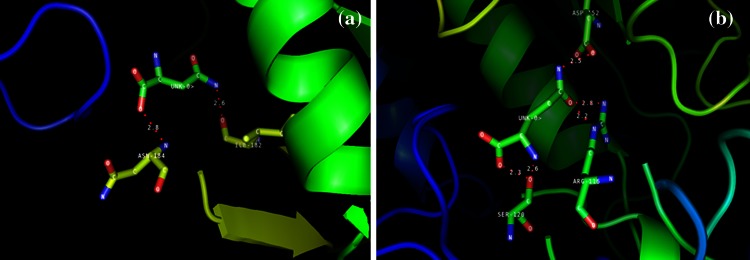
Molecular Docking results of 1NNS with ligand substrates. **a** Binding with l-asparagine. **b** Binding with l-glutamine

When docking runs were carried out by HEX 8.0.0, 1NNS showed very high binding affinity (i.e., high E total value) with l-Asn and l-Gln resulting in less free energy of −160.89 and −165.60 kJ/mol, respectively. Results obtained by HEX were validated by PatchDock webserver followed by refinement of obtained results with FireDock server. Outcomes strongly supported the previous results with a very good binding efficiency between receptor and ligands with the calculated global energy values of −22.72 and −21.46 kcal/mol for l-Asn and l-Gln, respectively, (this value is considered to be related to free binding energy) by FireDock server. Enzyme made inter molecular hydrogen bonds (shown in red color) with ILE-182, ASN-184 with l-Asn ligand and on the other side generated same inter molecular hydrogen bonds with ARG-116, SER-120, and ASP-152 when l-Gln as the ligand (Table 1). A summary of the binding energies by all the docking methods is described in Table 2. The mode of binding and cavity of ligands with receptor was investigated in PyMol molecular graphics viewer (Fig. 2). Crosschecking of the 1NNS binding sites in docking was done with active site prediction tool FT Site (Ngan et al. 2012; Brenke et al. 2009). The pooled results exhibited matching of most of the residues and thus the binding sites used in docking approach were well-defined (Refer to Online Resource 1). Present investigation results reveal that the nearly equal affinity of Elspar^®^ toward both the ligands strongly supports the asparaginase and glutaminase activities of the enzyme (Keating et al. 1993; Derst et al. 2000). Numerous adverse effects of l-asparaginase usage have also been stated, distant from severe immunogenic reactions (Kwon et al. 2009).

**Table 1:** Molecular docking results of 1NNS with ligand substrates

S. No.	Ligand	Residue: atom	Ligand atom	Bond length (Å)
1	l-Asparagine	ILE182: O	N_1_	2.6
		ASN184: N	O_3_	2.8
2	l-Glutamine	ARG116: NH_2_	O_1_	2.8
		ARG116: NE	O_1_	2.2
		SER120: OG	N_2_	2.6
		SER120: OG	O_2_	2.3
		ASP152: OD_2_	N_1_	2.5

**Table 2:** A summary of molecular docking results

Docking tool	Ligand	
	l-Asparagine	l-Glutamine
Hex 8.0	−160.89 kJ/mol	−165.60 kJ/mol
PatchDock and FireDock	−22.72 kcal/mol	−21.46 kcal/mol

## Molecular dynamics and simulations in water

**Fig. 3:**
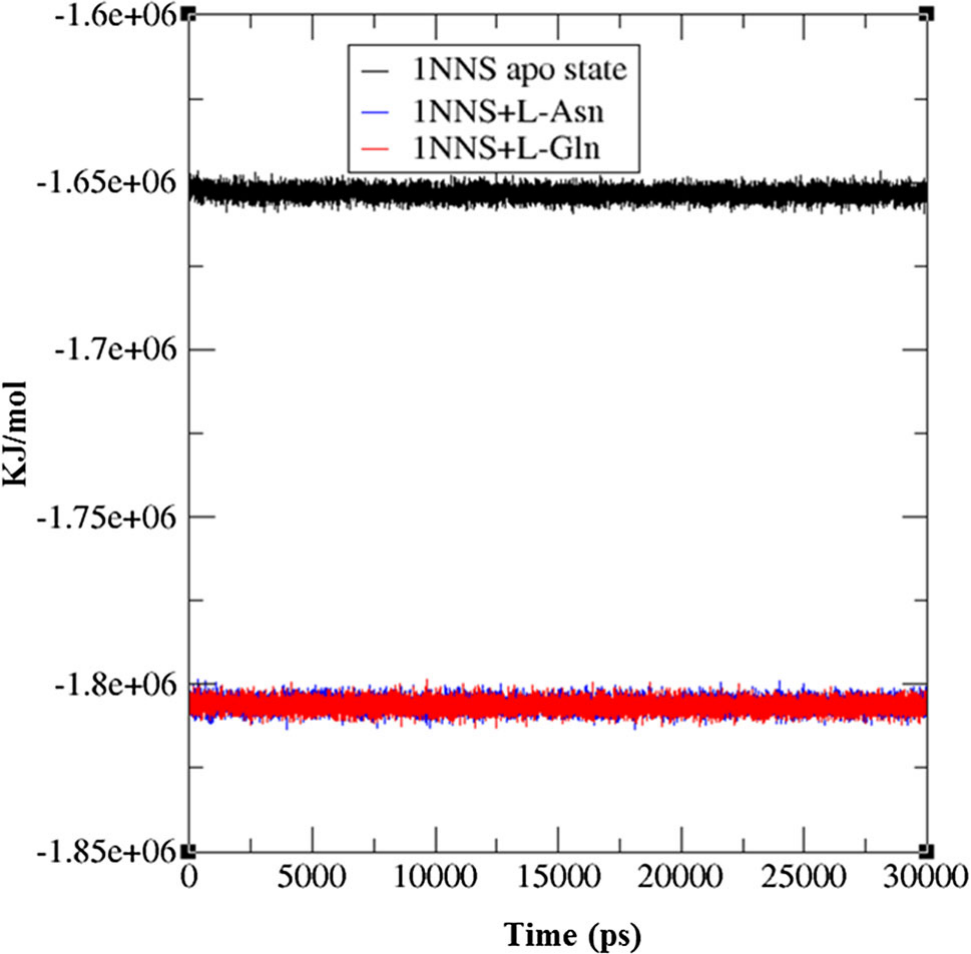
Total energy of apo state 1NNS and its docked complexes

**Fig. 4:**
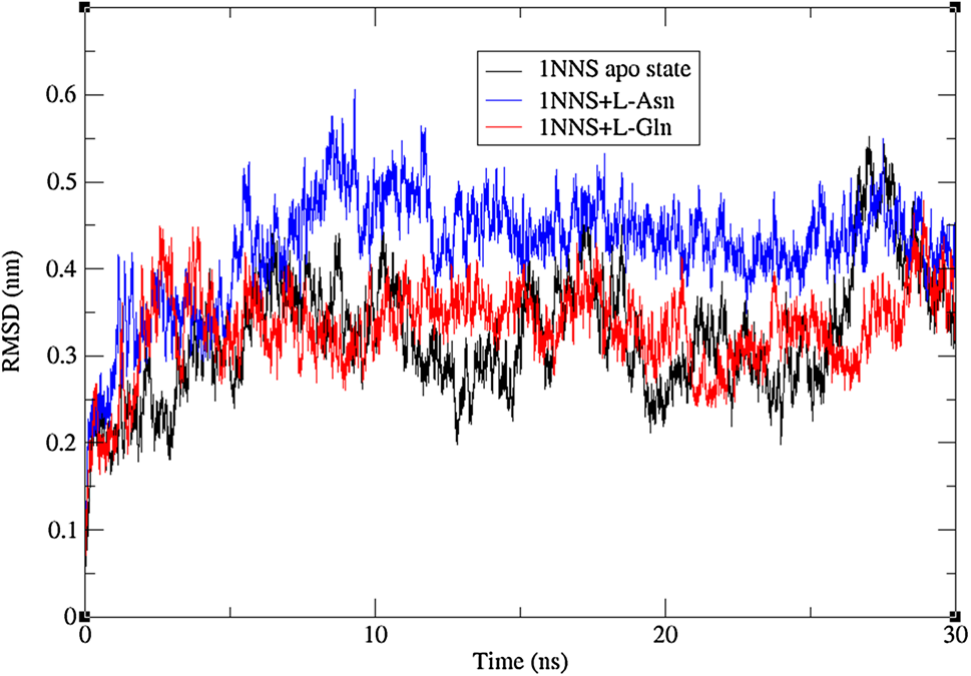
RMSD plots of backbone atoms during 30 ns simulations

**Fig. 5:**
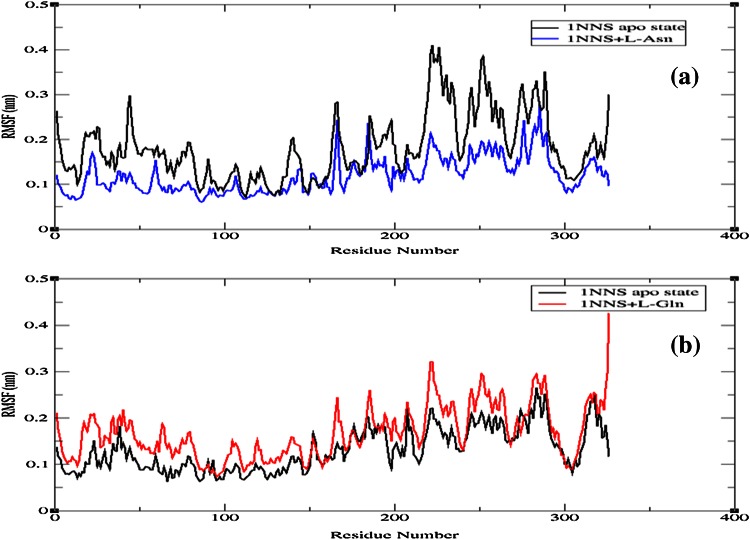
RMSF of Cα atoms along with RMSF of Cα atoms **a** 1NNS apo enzyme and 1NNS + l-Asn. **b** 1NNS apo enzyme and 1NNS + l-Gln

**Fig. 6:**
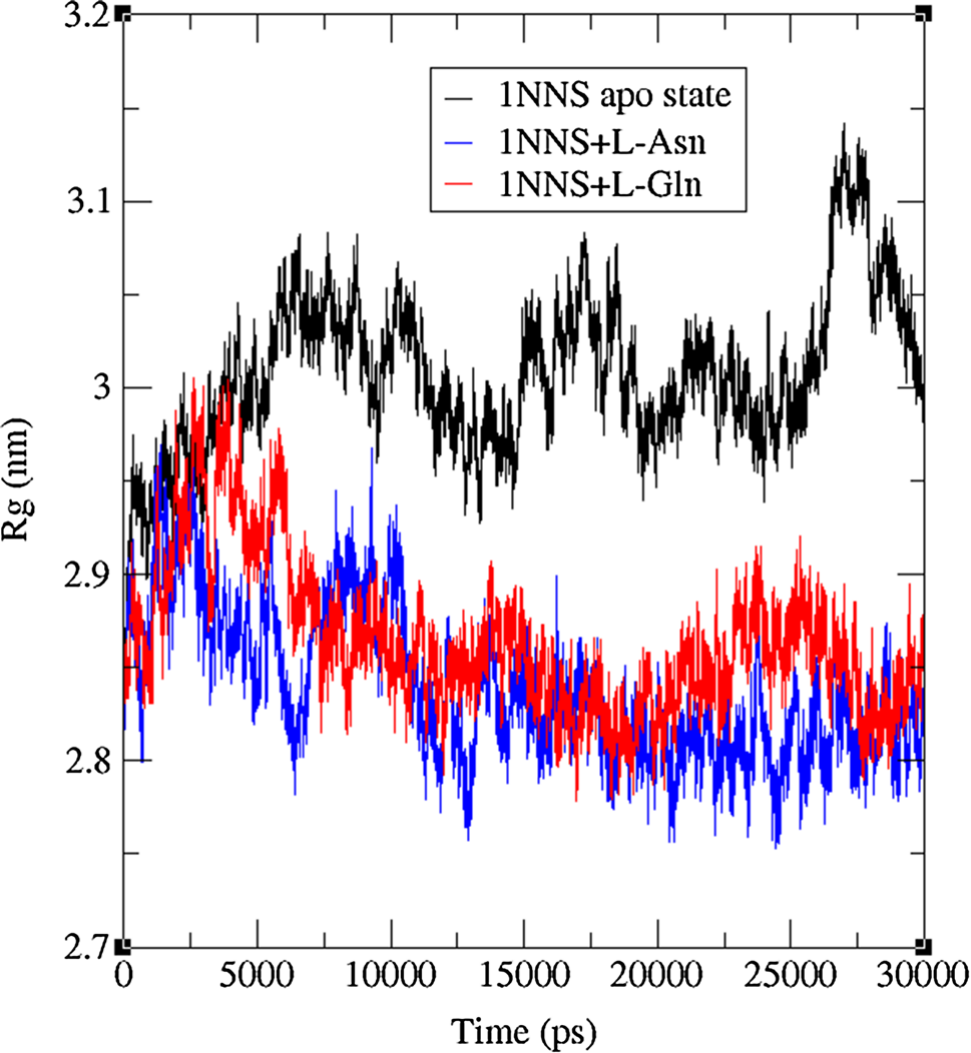
Radius of gyration plot during 30 ns simulations

**Fig. 7:**
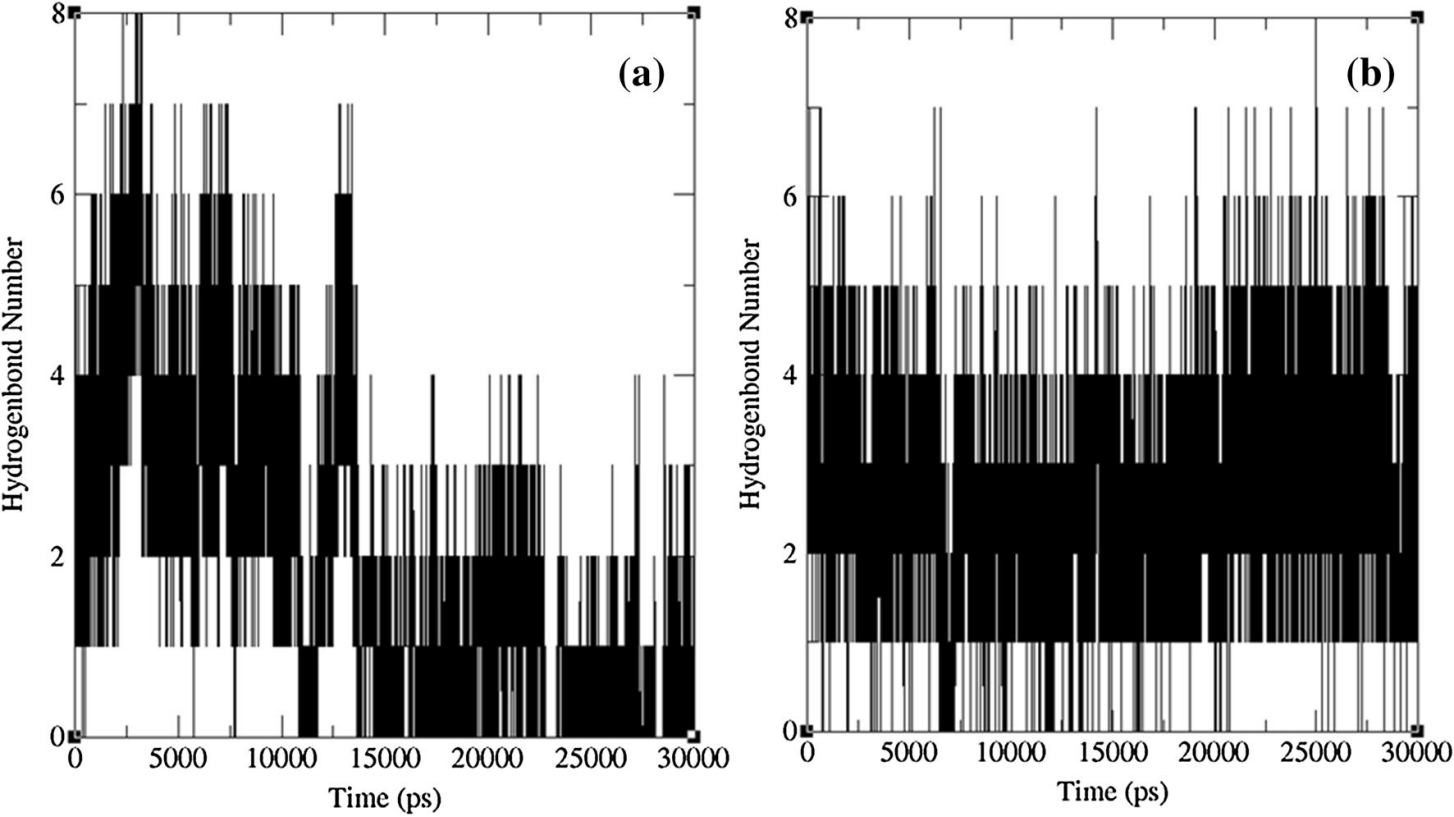
Inter-hydrogen bonding for docked complexes

To check the structural behavior of apo enzyme and docked complexes in the dynamic system, MD simulations were performed and the trajectory analysis was done using GROMACS utilities. The total energy found from g_energy results for apo enzyme, complex 1 and complex 2 was found to be −1,652,980, −1,805,840, and −1,806,110 kJ/mol, respectively, demonstrating that the models were energetically firm (Fig. 3). The structural convergence comprises terms like Root Mean Square Deviation (RMSD), Root Mean Square Fluctuation (RMSF), and Radius of Gyration (*Rg*). The g_rms based results with backbone atoms (Fig. 4) exhibited initial equilibration up to 5.5 ns and the structure underway to converge later and achieved stability with a RMSD between 0.2 and 0.5 nm with a peak after 26 ns for 1NNS in apo state, whereas the docked complex 1 attained equilibration at 10 ns. After the initial equilibration, it was stable throughout the trajectory oscillating between RMSD values of 0.4 and 0.5 nm which were a little higher than apo state enzyme. Although complex 2 initially followed apo state enzyme, it achieved a stable conformation throughout the simulation soon after the first 4 ns with an average backbone RMSD between 0.26 and 0.42 nm. By showing much higher consistency throughout the entire trajectory, docked complex 2 confirmed its stable conformation over both the apo state enzyme and complex 1. The examination of g_rmsf results presented the oscillations with Cα atoms with respect to residues. Results of g-rmsf analysis revealed a few fluxes in Cα atoms with respect to apo enzyme residues. As per RMSF plot residues of complex 1 are stable (Fig. 5a) with a few peaks with RMSF value of more than 0.25 nm compared to complex 2 (Fig. 5b) where many residues are oscillating around 0.3 nm along with one high peak at the last one with 0.4 nm. The comparison of RMSF outcomes showed minor variations in ligand binding sites (Table 3) and their effect on complex formation. Radius of gyration (*R*_g_) describes overall spread of molecule and was calculated using g_gyrate tool of GROMACS. Continuous drifts in gyration radii for apo state enzyme in the range of nearly 2.9 and 3.15 nm describe the dynamic nature of 1NNS. Docked complexes are better in terms of conformational stability with constant and low *R*_g_ values authorizing their better folding (Fig. 6) than unbound enzyme. Complex 1 was stable during entire MD run after 13 ns with no further drifts till end, and the second complex was stable only after 6 ns. The hydrogen bonding interactions between the docked complexes confirmed the hydrogen bonding pattern throughout 30 ns simulations (Fig. 7). Inter-hydrogen bond interactions pattern was clearly explained by the binding of l-Asn (Fig. 7a) and l-Gln (Fig. 7b) in the apo state initially with more number of hydrogen bonds which were reduced to 2–3 after the change in conformation due to binding after 12 ns simulations. Although MD simulation results were well correlated with molecular docking results with two to five hydrogen bonds seen in the dynamic state, surprisingly loss of hydrogen bonds at the end of simulations for complex 1 was observed. Overall, hydrogen bonding interactions revealed the affinity of ligand molecules with *E. coli* l-asparaginase and its dual activity. An overall result from this computational study confirms the dual functionality of the *E. coli* l-asparaginase enzyme. The instability of the enzyme upon the interaction with the ligand substrates based on RMSD, RMSF, and H-bond analysis suggests the need for identifying a new and stable l-asparaginase enzyme from diverse source with similar therapeutic effects for better curing of ALL.

**Table 3:** Comparison of RMSF values from MD simulations

S. no.	Residue	RMSF (nm)		
		1NNS in apo state	Complex 1	Complex 2
1	ARG116	0.0875	–	0.1016
2	SER120	0.0902	–	0.1305
3	ASP152	0.1667	–	0.1568
4	ILE182	0.1372	0.1295	–
5	ASN184	0.1684	0.2356	–

## Conclusion

l-asparaginase is one of the most attractive enzymes in cancer research. Investigational studies on *E. coli* sustained it as one of the therapeutic agents for ALL. In the present computational study, ligand binding pattern on 1NNS with l-Asn and l-Gln using molecular docking explained the binding mechanism at molecular level. Binding cavities and the key residues in binding were identified based on the docking results. MD simulations of complex 1 for 30 ns run confirmed the kinship of l-Asn in the dynamic system with less stability than complex 2. Overall findings strongly supported the bi-functional nature of the enzyme drug. Many conformational changes were observed with 1NNS structure due to the ligand binding. Results of this in silico studies favor added extensive structural research on l-asparaginase toward scheming of potential inhibitors that can be used in effective treatment of ALL.

## Electronic supplementary material

Below is the link to the electronic supplementary material.

Supplementary material 1 (PDF 284 kb)
