# Supplementary material for: Molecular dynamic simulations of Escherichia colil-asparaginase to illuminate its role in deamination of asparagine and glutamine residues
Source: 3 Biotech. 2015 Dec 29;6(1):2. doi: 10.1007/s13205-015-0339-9 (PMC4695448; doi:10.1007/s13205-015-0339-9)

1NNS binding Sites by FT Site Tool

Site 1 Residues	Site 2 Residues	Site 3 Residues
SER A 120	SER A 19	PHE A 127
SER A 122	ALA A 20	ASN A 131
ALA A 123	THR A 21	ASP A 152
PRO A 126	LYS A 22	ILE A 182
PHE A 127	SER A 23	HIS A 183
SER B 120	ARG A 116	ASN A 184
SER B 122	PRO A 117	GLY A 185
ALA B 123	SER A 120	ALA B 20
PRO B 126	MET A 121	THR B 21
PHE B 127	PHE B 127	LYS B 22
	ASN B 131	SER B 23
	ASP B 152	ARG B 116
	ILE B 182	PRO B 117
	HIS B 183	SER B 120
	ASN B 184	MET B 121
	GLY B 185	

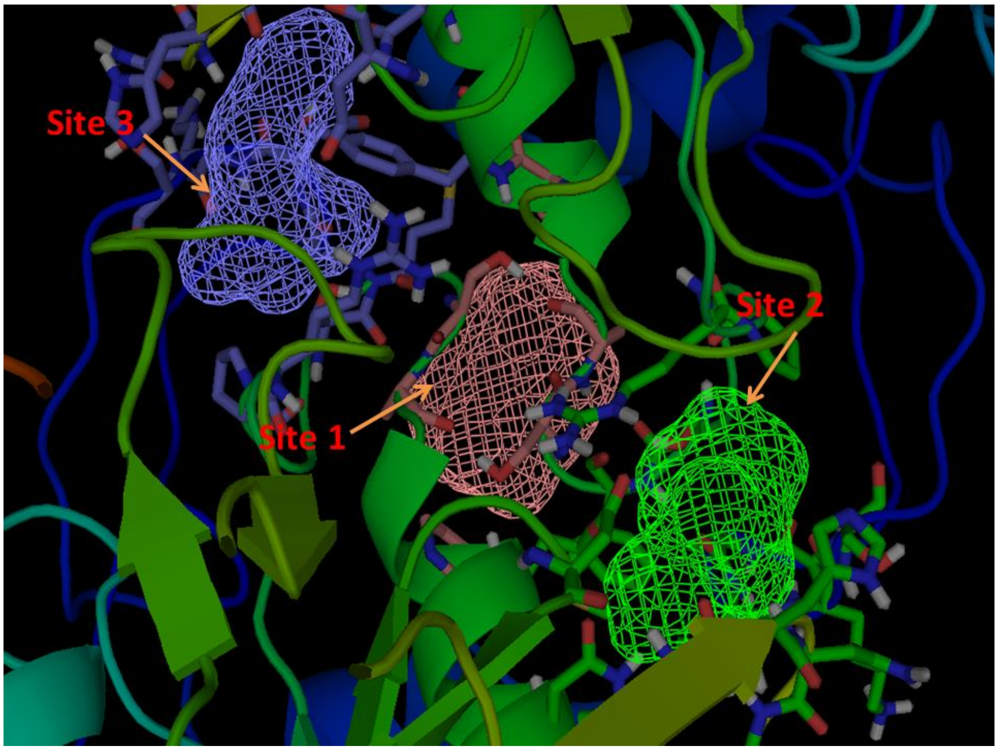

Supplement: Supplementary file 1 — Supplementary material 1 (PDF 284 kb) [file 13205_2015_339_MOESM1_ESM.pdf]
